# ‘Nutrition is out of our control’: soldiers’ perceptions of their local food environment

**DOI:** 10.1017/S1368980019001381

**Published:** 2019-06-21

**Authors:** Chizoba L Chukwura, Theresa Jackson Santo, Clarice N Waters, Anne Andrews

**Affiliations:** 1 ICF, Rockville, MD, USA; 2 Oak Ridge Institute for Science and Education (ORISE), Oak Ridge Associated Universities (ORAU), Former Participant in support of US Army Public Health Center, Health Promotion and Wellness Directorate, Public Health Assessment Division, ORAU, Belcamp, MD, USA; 3 US Army Public Health Center, Health Promotion and Wellness Directorate, 8977 Sibert Road, Aberdeen Proving Ground, MD 21010, USA; 4 US Army Office of The Surgeon General, Health & Wellness, Falls Church, VA, USA

**Keywords:** Food preferences, Environment and public health, Qualitative research, Military facilities, Environment design

## Abstract

**Objective:**

To explore the perceptions of soldiers participating in a US Army Office of The Surgeon General’s worksite health promotion programme (WHPP) on the local food environment within their campus-style workplace.

**Design:**

Focus groups were conducted to evaluate the perceived effectiveness of the WHPP implementation. Further exploration of focus group data through thematic analysis focused on perceived contributions of the military campus-style food environment to soldiers’ nutrition behaviours.

**Setting:**

Three US Army installations located in the continental USA.

**Participants:**

Active duty soldiers (*n* 366) participating in one of the fifty-eight focus groups.

**Results:**

Soldiers shared a common belief of self-discipline and personal responsibility as the foothold to nutrition behaviour change. Soldiers described aspects of the military campus-style food environment as factors impeding achievement of optimal nutrition. Collectively, soldiers perceived the proximity and density of fast-food restaurants, lack of healthy alternatives on the installation and the cost of healthy food as inhibitors to choosing healthy foods. Overwhelmingly, soldiers also perceived time constraints as a factor contributing to unhealthy food choices.

**Conclusions:**

Although nutrition behaviour is individually driven, soldiers perceived the military campus-style food environment inhibits healthy decision making. Nutrition programming in military WHPP must integrate food environment changes to improve soldiers’ nutrition behaviour outcomes. Applicable to the military, food choice behaviour studies suggest environmental changes must be appealing to young adults. Considerations for environmental changes should include an increased portion size for healthy options, broadened use of soldiers’ daily food allowances on local produce and increased availability of grab-and-go options.

For nearly three centuries, the US Armed Forces focused primarily on defending the nation’s borders from adversaries while fighting an internal struggle against health risks that endanger its service members’ lives, bear down its strength and consume its resources. In recent decades, highly prevalent threats to the health and overall readiness of the fighting force, and in turn the security of the nation as a whole^(^ ^1^ ^–^ ^3^ ^)^, include health risks caused by being overweight and obese. Being overweight has become a common medical disqualifier for military service^(^ ^1^ ^,^ ^4^ ^–^ ^6^ ^)^ and approximately 1200 first-term enlistees are medically discharged from the military each year due to weight-related health problems^(^ ^3^ ^–^ ^7^ ^)^. The percentage of active duty military personnel classified as obese rose from 5 % in 1995 to nearly 15 % in 2015^(^ ^1^ ^,^ ^8^ ^,^ ^9^ ^)^. This is consistent with reported national obesity trends of adults in the general civilian population, where the prevalence increased by approximately 10 % between 1999–2000 and 2015–2016^(^ ^10^ ^)^. However, the data indicate obesity prevalence in the general adult population is two and a half times greater than in active duty military personnel^(^ ^10^ ^)^. The 2005 Department of Defense (DoD) Health Related Behaviors Survey (HRBS) of Active Duty Military Personnel associated soldiers’ weight gain with poor eating patterns and inadequate consumption of fruits, vegetables and whole grains^(^ ^11^ ^)^. The 2014 HRBS^(^ ^12^ ^)^ results indicated one of every eight soldiers consumed the national daily recommended servings of fruits or vegetables.

In response to these trends, the military placed greater emphasis on promoting healthy eating behaviours. The DoD health promotion instruction (DoD Instruction 1010.10) recognizes the need for the military to design initiatives to influence the health decisions and behaviours of personnel and other beneficiaries^(^ ^13^ ^)^. Guided by this instruction, military services established worksite health promotion programmes (WHPP). In the non-military work environment, WHPP aim to protect and promote worker health, reduce health-related costs, and potentially improve worker health, well-being and productivity^(^ ^14^ ^)^. In the military, WHPP have an added aim of improving soldiers’ battle readiness and resilience. This instruction led to an Army-specific regulation that prioritized promoting healthy dietary habits as a focal area of support in WHPP^(^ ^15^ ^)^. Army regulation on nutrition standards and education further instructed nutrition-focused WHPP to adopt the US Department of Agriculture’s MyPlate nutrition standards to mitigate the rise in soldiers’ poor eating habits^(^ ^16^ ^,^ ^17^ ^)^.

Therefore, the Army Office of The Surgeon General developed a WHPP that targeted soldiers’ sleep, activity and nutrition (SAN) behaviours and piloted the programme for six months starting in September 2013. The Army WHPP focused on improving soldiers’ SAN behaviours in order to enhance their operational readiness and resilience through leadership engagement; a health communication campaign promoting SAN; provision of fitness trackers to all soldiers in participating units to facilitate self-monitoring of SAN; and squad leader-facilitated small group health education sessions. Many WHPP focus primarily on knowledge dissemination and overlook key factors heavily influencing behavioural processes, such as the built environment^(^ ^18^ ^)^. When the Army WHPP pilot was evaluated in March 2014, some participants reported increased SAN knowledge but few reported behaviour changes^(^ ^19^ ^)^. This finding is not unique to military populations. A systematic review of literature in tertiary education settings showed that single-intervention strategies aimed at food labelling and promotional materials for healthy foods were mildly successful in modifying diet behaviours^(^ ^20^ ^)^. However, when these educational interventions were coupled with an increase in healthy food availability and accessibility, dietary changes were more prevalent^(^ ^20^ ^)^. Other studies suggest that positive perceptions of access to healthy foods is highly correlated with dietary behaviour change^(^ ^21^ ^,^ ^22^ ^)^. Changing the physical environment to influence conscious and unconscious behaviours will consequently increase healthy behaviour^(^ ^20^ ^–^ ^23^ ^)^.

Leading public health agencies such as the WHO, the Institute of Medicine, the International Obesity Task Force and the Centers for Disease Control and Prevention stress the need to incorporate the environment and policy in health programme planning to improve nutrition behaviours^(^ ^23^ ^)^. Although the significance of considering the environment in health promotion planning for the general population is understood, limited evidence is available in this area as it pertains to the military populations or the Army specifically. Understanding soldiers’ perspectives of their food environment is essential to plan a comprehensive WHPP that will change nutritional behaviour. Therefore, the present exploratory analysis of qualitative evaluation data aimed to gain a better understanding of active duty Army soldiers’ perceptions of their food environment and enhance the body of evidence regarding perceived effects of military environmental factors on eating behaviours.

## Methods

At the conclusion of the six-month pilot, the Army Public Health Center conducted a post-implementation programme evaluation of the Army WHPP from March to May 2014 set in campus-style environments, including soldiers’ domiciles, various work environments, and commercial and university-inspired dining facilities. The objective of the Army WHPP pilot evaluation was to determine the programme’s impact on soldiers’ SAN behaviours and to provide recommendations to ensure the programme and its activities are maximally effective, efficient and sustainable prior to Army-wide expansion. The present paper reports the findings from an exploratory analysis of a sample that participated in the Army WHPP pilot (subsequently referred to as ‘WHPP’) and its evaluation.

### Population and sample

**Fig. 1:**
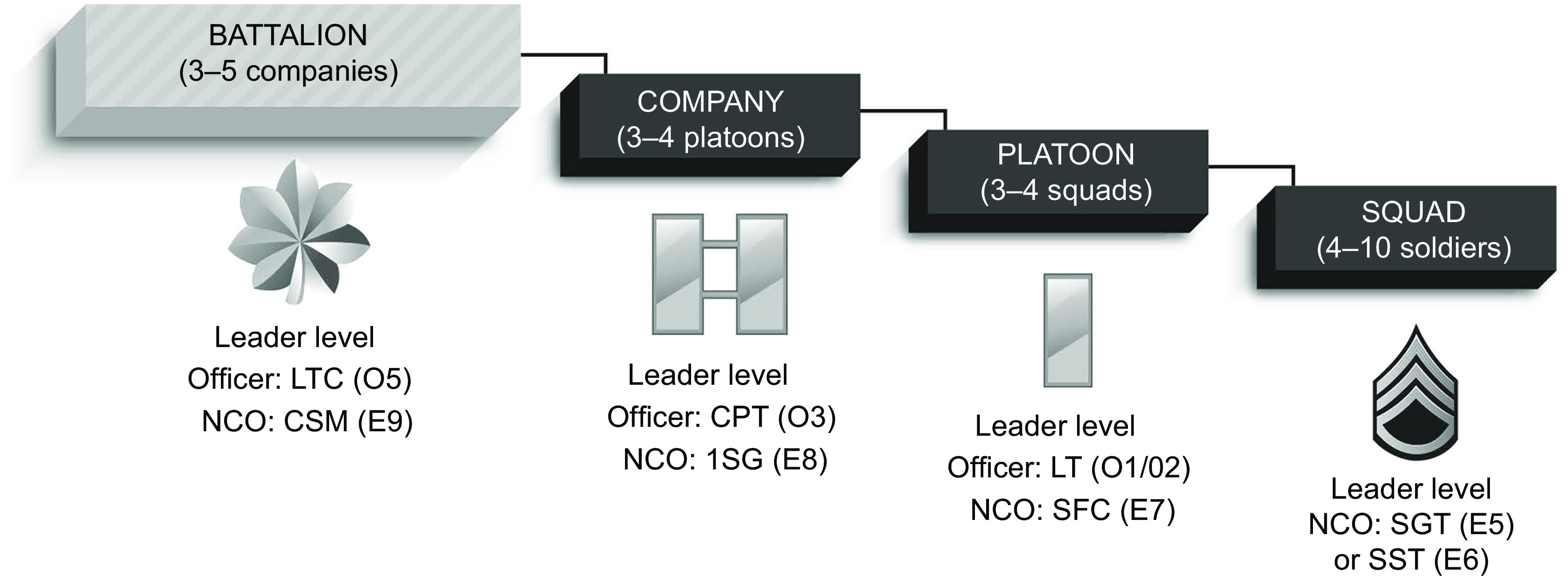
US Army battalion organization structure (LTC, Lieutenant Colonel; NCO, non-commissioned officer; CSM, Command Sergeant Major; CPT, Captain; 1SG, First Sergeant; LT, Lieutenant; SFC, Sergeant First Class; SGT, Sergeant; SSG, Staff Sergeant)

Three battalion-sized units (one combat support squadron, one combat sustainment support battalion and one infantry battalion), each comprising approximately 400–1000 soldiers, participated in the WHPP and its evaluation activities (see Fig. 1 for a depiction of an Army battalion’s organizational structure). The participating combat support squadron and combat sustainment support battalion were cavalry and logistics units, respectively. Each battalion was located on a different Army installation (may be referred to as a ‘post’ or ‘base’) within the continental USA. Army leadership purposely selected these battalions because they were reflective of the three primary battalion types of the Army. One of the objectives of the WHPP pilot was to evaluate the feasibility of Army-wide implementation, thus including units with different missions was critical to ensuring that the findings could apply across various Army unit types. In total, approximately 2000 demographically representative soldiers participated in the WHPP pilot^(^ ^24^ ^)^.

Battalion-level senior leadership at each site selected soldiers to participate in focus groups. Recruitment targets were set at a minimum of eighteen groups per site to allow for saturation of defined categories of interest among each represented soldier rank segment. Table 1 outlines the different rank groups and positions. Senior-level enlisted leaders and officers were grouped together in the position of ‘Platoon Leaders and Higher’, while the position of ‘Unit Support’ refers to unit medical and military treatment facility (medical clinic) staff.

**Table 1:** Soldier rank segments for targeted battalion unit positions

Targeted audience/unit position	Enlisted/officer level	Soldier rank segment
Squad Members	Junior Enlisted	E1–E4 (PVT–SPC)
Squad Leaders	Senior Enlisted	E5–E6 (SGT–SSG)
Platoon Leaders and Higher	Senior Enlisted and Officer	E7–E9 (SFC–SGM/CSM)O1–O5 (LT–LTC)
Unit Supports*	Various	Various

PVT, Private; SPC, Specialist; SGT, Sergeant; SSG, Staff Sergeant; SFC, Sergeant First Class; SGM, Sergeant Major; CSM, Command Sergeant Major; LT, Lieutenant; LTC, Lieutenant Colonel.

* Participants categorized as ‘Unit Supports’ include unit medical, medical treatment facility and battalion assets; these participants included soldiers of various ranks.

### Data collection

**Fig. 2:**
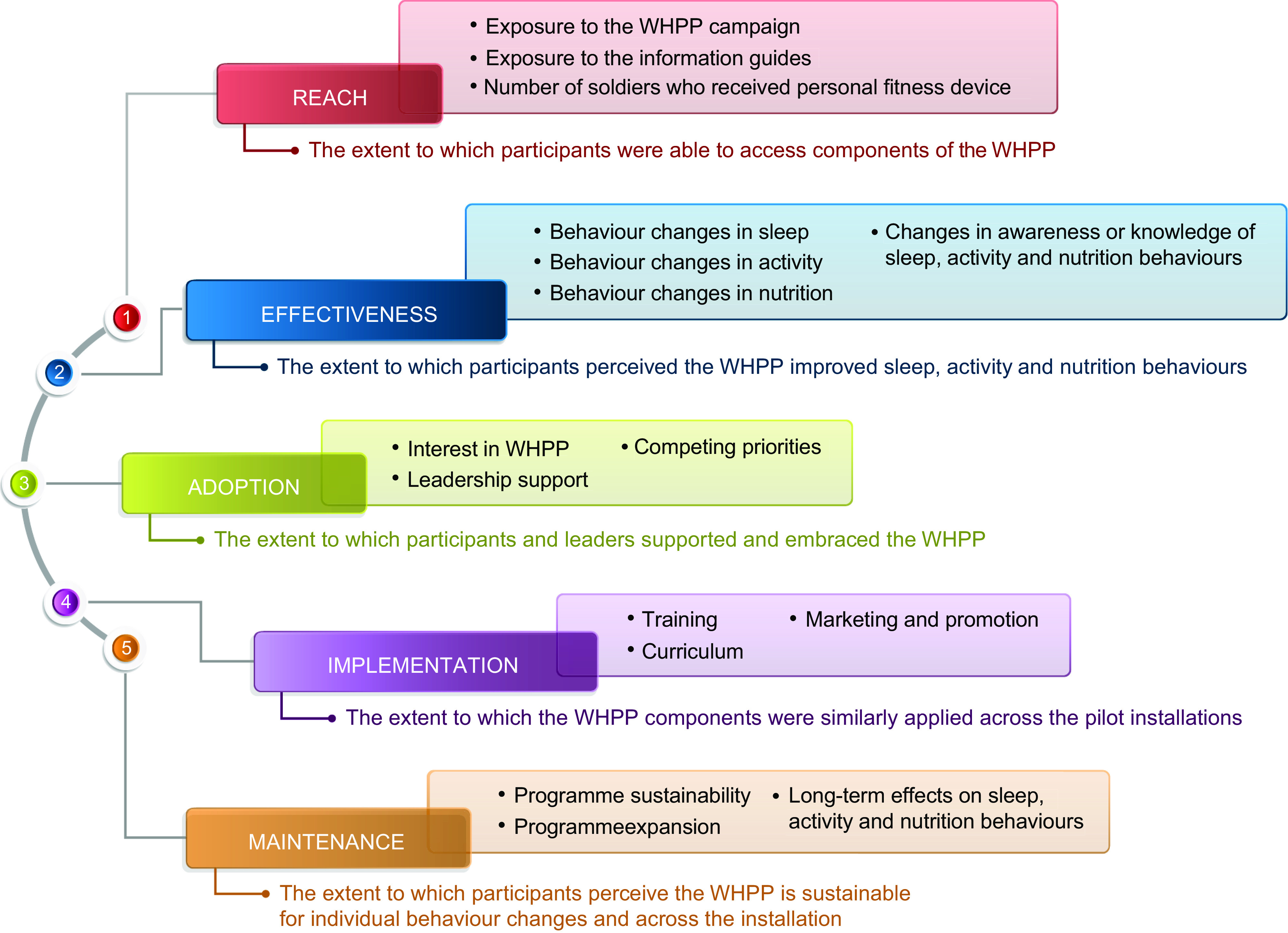
Discussion topics guided by the RE-AIM (Reach, Effectiveness, Adoption, Implementation and Maintenance) framework (WHPP, worksite health promotion programme)

Qualitative data were collected as part of the larger WHPP pilot evaluation guided by the RE-AIM (Reach, Effectiveness, Adoption, Implementation and Maintenance) framework (Fig. 2)^(^ ^25^ ^,^ ^26^ ^)^. Evaluators operationally defined each RE-AIM construct to steer the development of two primary focus group guides consisting of twelve to sixteen open-ended questions. Figure 2 outlines discussion topics for each of the RE-AIM constructs within the guides; evaluators developed questions and associated probes to assess these constructs. For example, within the effectiveness construct, questions included: ‘Have you noticed any changes in your personal sleep knowledge or habits over the last six months? If so, could you describe those changes?’ and ‘What about changes in your unit Goverall with regard to sleep, activity and nutrition?’ Focus groups were segmented by rank and role to minimize the risk of dominating participants due to the hierarchical structure in the military and ensure comfort with sharing opinions^(^ ^27^ ^,^ ^28^ ^)^.

An appointed team of evaluators trained in qualitative data collection techniques and protocols (two moderators, two note-takers and the lead project officer) visited each WHPP site for one week between March and May 2014. Participants were provided information sheets describing the purpose of the focus groups and were informed their participation was voluntary. All participants verbally consented to participate to maintain participants’ confidentiality. With participants’ additional consent, each discussion was audio-recorded on a primary and secondary digital audio recorder. Evaluators conducted fifty-eight focus groups, lasting 60–90 min each. Facilitators and note-takers completed end-of-group summaries documenting group dynamics, emergent themes and key discussion areas.

### Data management and analysis

A contracted service transcribed audio files verbatim and redacted identifying information. The evaluation team reviewed transcripts for accuracy and completeness. Two qualitative data analysts manually open-coded two randomly selected transcripts and reviewed literature on the RE-AIM framework to develop the initial codebook in NVivo 9 (QSR International, Doncaster, VIC, Australia). The analysts then independently coded four randomly selected transcripts in tandem using NVivo to ensure inter-coder reliability and compare coding consistencies. Once a reliability of 80 % was reached, the remaining fifty-two transcripts were split evenly between the two analysts and were independently coded using the initial codebook. Subsequent rounds of coding interspersed with debriefing sessions involving the evaluation team led to codebook refinements.

**Fig. 3:**
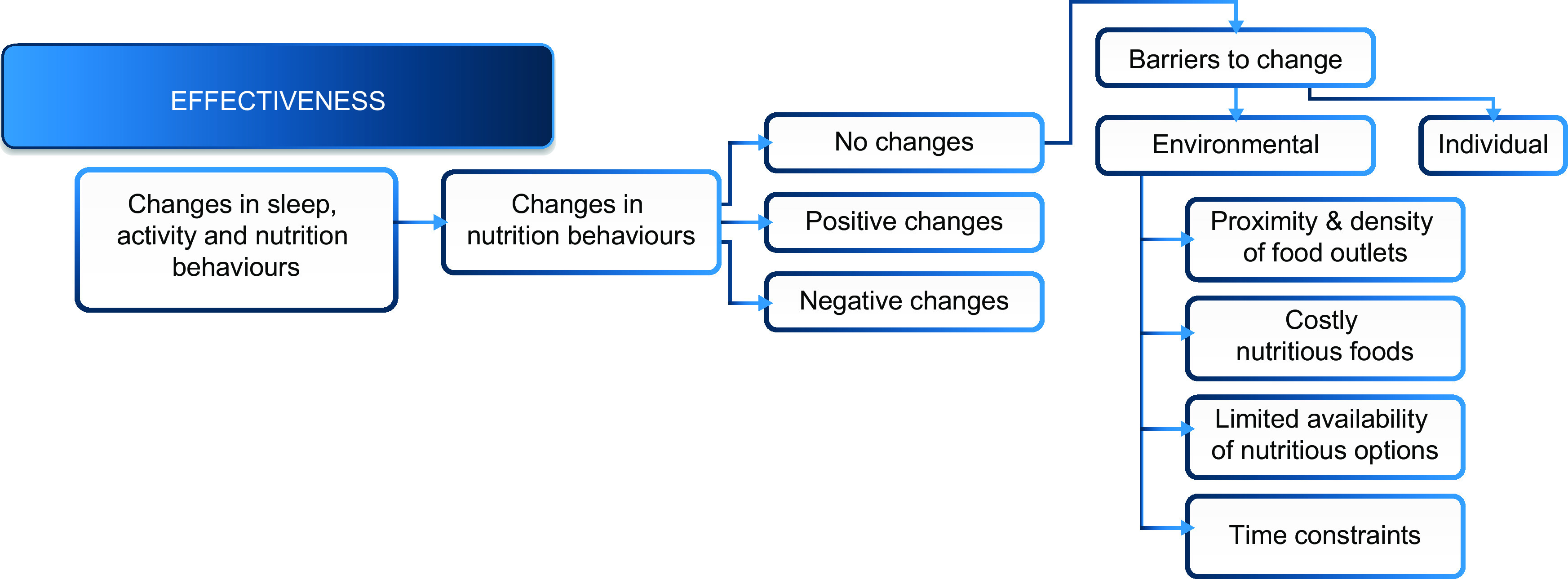
Major themes related to barriers in the Army food environment revealed by focus group discussions with active duty soldiers (*n* 366) at three US Army installations located in the continental USA, March–May 2014

Analysts conducted thematic explorations to determine themes related to the Army food environment. Within the effectiveness construct, analysts further explored recurring codes related to changes in nutrition behaviours. Specifically, for the groups in which participants indicated there were no changes in their nutrition behaviours (i.e. an indicator that the WHPP was ineffective in this domain), analysts identified emergent barriers to nutrition behaviour change. Barriers were themed as environmental (those factors external to an individual that impeded behaviour change) and individual (knowledge and attitudes unique to individuals that impeded change). Within the environmental barriers, themes were identified based on two criteria: (i) the highest frequencies of recurring codes; and (ii) themes that were salient across demographics, installation and battalion type. Figure 3 illustrates the relationship between codes and the identified major themes related to the Army food environment. Individual barriers are not presented in the current paper.

Illustrative quotes within codes relevant to Army food environment barriers were extracted to support key themes. Quotes presented in the results are cited by type of battalion unit and the participants’ position (Table 1). Quotes representing all leaders, but not squad members and unit supports, are denoted as ‘leaders’.

## Results

### Demographics

As a result of recruitment efforts, 366 soldiers participated in the focus groups. The sample consisted of Squad Members (44 %), Squad Leaders (27 %), Platoon Leaders and Higher (21 %) and Unit Supports (7 %). Participants were predominantly male with a mean age of 28·0 (sd 7·5) years; the logistics unit had the highest mean age (30·2 (sd 7·9) years), followed by the cavalry unit (27·8 (sd 7·5) years) and the infantry unit (26·4 (sd 6·7) years). Most participants reported having at least some college education, and the majority of participants reported serving at least six months in their current unit (Table 2). There were some differences in participant distribution across sites such that the infantry battalion unit had a larger amount of squad members compared with other battalion units.

**Table 2:** Demographic characteristics of the focus group participants; active duty soldiers (*n* 366) at three US Army installations located in the continental USA, March–May 2014

	Cavalry		Infantry		Logistics		All units	
	*n*	%	*n*	%	*n*	%	*n*	%
Total	98	100	149	100	114	100	363	100†
Gender								
Male	96	98	145	97	86	75	327	91
Female	2	2	4	3	28	25	34	9
Rank								
Squad Members	44	45	73	48	41	36	158	44
Leaders	41	42	70	46	63	56	174	48
Squad Leaders	28	29	29	19	41	36	98	27
Platoon Leaders and Higher	13	13	41	27	22	20	76	21
Unit Supports*	9	9	8	5	10	9	27	7
Time with unit								
Less than 6 months	19	19	26	17	32	29	77	21
6 months to 1 year	21	21	45	30	20	18	86	24
1 to 2 years	23	24	42	28	34	30	99	28
More than 2 years	35	36	36	24	26	23	97	27
Education level								
High school or GED	27	28	64	43	23	21	114	32
Some college	35	36	43	29	47	42	125	35
2-year college degree	7	7	10	7	11	10	28	8
4-year college degree	20	20	27	18	21	19	68	19
Graduate/advanced degree	8	8	5	3	9	8	22	6
Other	1	1	0	0	1	1	2	1

GED, General Equivalency Diploma.

* Participants categorized as ‘Unit Supports’ include unit medical, medical treatment facility and battalion assets.

† Three focus group participants orally consented but did not provide demographic information; therefore, overall total *n* reported in this table is less than the total number of focus group participants.

### Focus group findings

From the overall pilot programme evaluation, soldiers in about half of the focus groups reported positive changes in their nutrition knowledge and awareness. Soldiers in approximately one-third of the groups reported making positive nutrition behaviour changes. However, soldiers in the majority of focus groups indicated that they made no positive nutrition behaviour changes and nearly two-thirds of groups reported barriers to nutrition behaviour change. Commonly identified barriers to nutrition behaviour change were associated with the community, organizational and consumer aspects of the local food environment.

A common thread vocalized among all soldier ranks in regard to achieving optimal nutrition was ‘nutrition is out of [their] control’. Soldiers often portrayed their local food environment as grounds for their inability to make a nutrition behaviour change. Leaders and squad members alike identified multiple environmental conditions that were barriers to achieving optimal nutrition. Along with the environmental conditions, soldiers specified ‘limited time’ as an added barrier to eating healthily. Four key themes related to effective nutrition behaviour change are presented in Table 3 and more details of the specific barriers are presented below.

**Table 3:** Key themes identified as barriers to healthy eating within Army food environments, stratified by soldier rank, as revealed by focus group discussions with active duty soldiers (*n* 366) at three US Army installations located in the continental USA, March–May 2014

Key themes	Platoon Leaders or Higher	Squad Members or Leaders
Proximity and density of fast-food outlets	High density of fast-food outlets Fast food is available but lacking nutritious options	Close proximity of fast food is convenient
Cost of nutritious foods	Healthy options cost too much Value satiety over nutrition	Healthy options cost too much Small portions are not cost-effective
Limited nutritious options available	DFAC have poor food quality	DFAC have small portion sizes Lack of healthy vending options
Time constraints	Short lunch period Long lines at DFAC	Limited time for meal preparation

DFAC, dining facility.

#### 1. Costly nutritious foods impeded positive changes to nutrition behaviour

Across sites, leaders and squad members expressed challenges with nutrition behaviour change due to the perceived high expense of healthy foods or better cost value of less nutritious options. The logistics battalion soldiers mentioned having difficulty with nutrition in relation to high food costs less frequently compared with the other battalions. When the cost of food was mentioned, it was repeatedly stated in relation to a soldier’s income or budget constraints. Some soldiers do not receive a food allowance and are expected to eat in unit dining facilities (DFAC). The DFAC provide meals at a fixed price. While they can eat somewhere else, they are not provided a subsistence allowance to offset the cost. Cavalry squad members described their struggle with affording healthy food options on a small budget or food allowance plan:

‘[Attaining optimal nutrition] can possibly happen … [if] you have dependants and they [the Army] actually give[s] you money to go to the store and buy your own food … [but] it’s hard … if you’re a single soldier, we don’t get that $300, $350 to just go buy our own food.’

Healthy eating discussions in a few focus groups consisting of mainly squad leaders described soldiers’ valuing satiation over nutrition with respect to the relative cost of food. In those discussions, some soldiers expressed beliefs that the DFAC portioned nutritious foods in too small quantities. An infantry squad leader gave an account of how food prices and portion sizes influenced his breakfast decisions:

‘A half scoop of hash [in the DFAC] … that doesn’t do anything for me. No wonder the kids want to go eat [national fast-food chain 1] or [national fast-food chain 2] or whatever for breakfast when they’re gonna get that kind of portion size. If I’m gonna pay $2·50, then I’m gonna go off post and spend an extra $2·50, and get something that’s actually gonna make me feel full when I’m done, instead of a half a scoop of eggs and just feel insulted when I leave.’ (Infantry, squad leader)

Many other soldiers, particularly squad members, expressed their shared frustration with the small DFAC portion sizes:

‘Most [of] the time they’re [the DFAC servers] trying to proportion it out equally amongst the people, so then you get like a small portion.’ (Infantry, squad members)

#### 2. Limited nutritious options in Army work environments made eating healthily seem unrealistic

Nearly all soldiers attested that the plethora of unhealthy food options on the installation was a barrier to nutrition behaviour change. In many work environments, vending machines with unhealthy options seemed to be the only choice for food purchase. Infantry battalion and cavalry squadron units reported more barriers with food vending options on their installations than the logistics battalion unit. Leaders and squad members spoke equally poorly of their respective installations’ DFAC. Both soldier rank groups described the perceived unappealing aspects of food at the DFAC by stating the DFAC primarily served processed foods or prepared otherwise healthy foods in large amounts of grease:

‘Some of them [food choices] are poorly cooked, poorly prepared, or … it’s not even healthy … for me I find that [healthy eating is] kind of not really realistic.’ (Infantry, squad member)

Leaders’ discussions showcased a sense of empathy towards lower-ranking soldiers for whom DFAC were their primary choice for food:

‘The DFAC is pretty much the only choice for my soldiers, and the DFAC a lot of times serves processed food, food that’s … just not very healthy food. Even if you go through and try to eat the healthiest you can, ’cause I know we all eat there whenever we have staff duty, and I’ve never been impressed.’ (Infantry, platoon leaders and higher)

Aside from the DFAC, most soldiers expressed frustration with the food selection in the field and during deployment, recognizing ‘that nutrition in the field goes out the window’. An infantry squad member noted that the food selection,

‘… may be different [depending on] where you are, if you have access [to nutritious foods], [or] if you have a good dining facility.’

The infantry squad member further explained that when food facilities are unavailable, soldiers are given pre-packaged, high-energy field rations,

‘… but if you are out … doing missions, out doing patrols, [or] you’re out in the field, you are going to get three meals ready-to-eat (MREs) a day, if that.’

While nearly all soldiers who participated in the focus groups have been deployed, the infantry soldiers commented on the lack of nutrition in the field and during deployment more than the combat support soldiers and described circumstances in which they could not receive three meal rations per day.

#### 3. The proximity and density of food outlets influenced soldiers’ food selection

Senior-level and squad leaders expressed frustration over the abundant availability of fast-food options on their Army installations. They perceived the high number of fast-food outlets lure soldiers to eat fast food:

‘… all these fast food chains, they’re everywhere, but they don’t offer any healthy options … Why is all that temptation there? Why do we have those food chains on this post?’ (Infantry, squad leaders)

As with the squad leaders, senior-level leaders voiced their frustration through a line of rhetorical questions: ‘Why don’t we have a healthier [option] – why do we have all these fast foods?’ The majority of lower-ranking soldiers from all sites primarily remarked that the convenience of fast-food options led them to make unhealthy choices and that they found the dearth of nutritious options problematic.

Many soldiers perceived fast-food restaurants located on the installations as convenient, acknowledging that the ease of accessibility influenced their choice to consume the unhealthy food options. Convenience, for some soldiers, was defined as an available food outlet requiring minimal food preparation and travel time to and from their respected worksites. A squad member from a logistics focus group commented that,

‘… a lot of people eat fast food just because of its convenience … because they don’t have time to go home for a meal.’

Infantry and cavalry soldiers depicted fast-food restaurants as relatively close to their work or duty station, specifying the proximity as either ‘right around the corner’ or ‘down the street’.

#### 4. Time constraints prohibited healthy food consumption

Soldiers in all ranks often mentioned their decision for eating poorly in connection with time constraints. Many soldiers depicted their time constraints as set by their environment, with one infantry squad leader identifying that it ‘boil[s] down to our jobs’. Many of the soldiers perceived that squad members are forced to choose unhealthy meals due to the coupled effect of the short time allotted for lunch and long lines at the DFAC. When limited by time,

‘… the soldiers say they don’t have enough time to go to the dining facilities for lunch and so what is available quick, [snap fingers] you know, quick lunch [snap fingers] access … [to] things like the gut trucks [food delivery trucks], the fast foods…’ (Calvary, unit supports)

Some soldiers noted during focus groups that they had been opting for unhealthy meals at home due to after-work responsibilities and limited time for meal preparation:

‘I mean, you get home from work, “Yeah, I’m going to look in my [WHPP] book and I’m going to see, alright, nutrition. What do I want to do for dinner?” Well, this says that I should probably eat this and this and this but I’ve got like laundry to do and I have to study for this test and I have to do a little extra PT [physical training] by myself and I’ve got to put together these slides and all this other stuff. So, I mean, let me just order a pizza.’ (Logistics, squad members)

## Discussion

The findings from the current exploratory analysis demonstrated that soldiers of all ranks experienced barriers to eating healthy, nutritious foods in Army campus-style environments. Focus group discussions revealed themes in which factors limiting healthy food choices were all associated with the Army environment: the lack of physically available nutritious options on the installation while there is an abundance of fast-food options; the perceived cost of healthy options; and time constraints. Soldiers described that these factors restricted their ability to choose healthy options and influenced their poor food choices. These findings are congruent with other published results from comparable studies in non-military populations on perceived environmental barriers to healthy nutrition behaviour change^(^ ^20^ ^–^ ^22^ ^)^.

A study by Glanz and colleagues^(^ ^29^ ^)^ ascertained five factors responsible for food choice (in order of importance): taste, cost, nutrition, convenience and weight control. Responses regarding the cost of nutritious foods from junior enlisted soldiers indicated they preferred satiation to the perceived higher expense of healthier options. Results from a food choice behaviour study indicated that young adults with low incomes, a demographic characteristic representing junior enlisted soldiers, found cost and convenience of utmost importance when selecting food^(^ ^29^ ^)^. The focus groups supported this conclusion and revealed price effects as a factor driving food choices among soldiers. Young males consider value for dollar as an important aspect in their food choices^(^ ^30^ ^)^. To this effect, many soldiers in our focus groups spoke of compromising healthy eating for satiety obtained from consuming low-cost, large-portioned, highly caloric meals at fast-food restaurants. Consistent with our soldiers’ responses, experimental studies have demonstrated the effectiveness of perceived lower cost and large serving sizes in influencing greater food consumption^(^ ^31^ ^)^. Food consumer behaviour research classifies the combination of price and portion size as the two major components in an individual’s perceived value of food^(^ ^29^ ^,^ ^31^ ^)^. Employing the same logic, several soldiers in the current evaluation attributed a higher value to fast-food options which they associated with satiation.

Proximity to food resources, limited transportation options and cost are commonly cited factors influencing impulse buys at fast-food restaurants by low-income individuals^(^ ^30^ ^,^ ^32^ ^)^. Two themes emerged, convenience and the abundance of fast-food outlets, which greatly influenced a soldier’s decision towards choosing unhealthy meals. In Boone-Heinonen *et al*.’s study^(^ ^30^ ^)^, low-income men who lived in an area of high fast-food restaurant density had increased consumption of fast food with poor diet quality. Similarly, many soldiers in our study expressed an inability to resist the temptation of unhealthy eating habits due to the abundance of fast-food options in close proximity to their workplace. Further, many soldiers noted they frequented fast-food establishments because they were available on the installation and required less time than eating in the DFAC. Consistent with the findings of our analysis, other studies have shown that time is a barrier to accessing healthy foods for individuals who work non-standard schedules^(^ ^33^ ^,^ ^34^ ^)^. Stringent schedules and busy lifestyles leave little time for soldiers to prepare and consume healthy meals. Mandatory physical training coupled with short breaks and long work hours make fast foods and pre-prepared foods at DFAC the more convenient option for soldiers. Long work hours and insufficient sleep due to increased workloads have been statistically linked to poorer access to health-promoting services and to increased risk of obesity and other physical ailments such as high blood pressure and diabetes^(^ ^33^ ^,^ ^35^ ^–^ ^37^ ^)^.

### Recommendations

Recognizing that individual behaviours are influenced by environmental factors^(^ ^38^ ^,^ ^39^ ^)^, the Centers for Disease Control and Prevention recommends that environments should support healthy choices by increasing the availability and accessibility of healthy food options^(^ ^40^ ^)^. With a mission to become ‘the soldier’s preferred dining choice’, Army DFAC currently offer more meal selections at a relatively low cost (~$US 4·62/meal)^(^ ^41^ ^)^. While healthy options are available within the DFAC, soldiers perceive DFAC portion sizes are small and lack appeal, particularly for healthy foods. To mitigate this perception, the Army could provide education to soldiers on ways to create a nutrient-balanced meal at current portion sizes to help soldiers feel satiated longer (e.g. high fibre, adequate protein) or consider making portion sizes larger for healthy foods and smaller for less nutritious options. The price reduction of nutrient-rich foods and price increase of less nutritious foods has been an effective strategy^(^ ^30^ ^,^ ^42^ ^)^ and could be considered on Army installations to promote purchasing healthy food options in a variety of environments (e.g. in vending machines, within convenience stores).

Other strategies to improve the availability and accessibility of healthy options are rooted in policy change. Similar to other national food assistance programmes, the Army could allow soldiers to use their basic daily food allowance to purchase local produce from community gardens and farmers’ markets on the installation or in close proximity to the installation in an effort to increase their fruit and vegetable consumption. The Army could also ensure that contracted food establishments follow DoD and national recommendations and guidelines restricting the allowable amount of unhealthy food offerings when on military installations. Similarly, the Army could restrict the amount of unhealthy food choices for food trucks to be permitted to operate within installation boundaries. Army regulation exists to maintain nutritional standards within Army-owned dining facilities^(^^16^ ^)^. However, national or regional chain food establishments and vending machines permitted, by contract, on the installation are usually not regulated by the Army to contractually restrict specific allowable food items. A federal precedent for reviewing food contractors (i.e. competitive foods) was established in the Healthy, Hunger-Free Kids (HHFK) Act at the end of 2010 for school nutrition guidelines. Section 305 of the HHFK Act added a new provision requiring state and local entities and their contractors participating in the programmes under the Richard B. Russell National School Lunch Act (NSLA) and the Children Nutrition Act of 1966 (CNA) to cooperate in studies and evaluations conducted by or on behalf of the US Department of Agriculture (Section 28 (c) of the NSLA, 42 USC. 1760(c))^(^ ^43^ ^)^. Such regulation to evaluate and review competitive or contracted foods on Army installations could result in necessary changes to their offerings and improve the availability of healthy options in close proximity to soldiers.

It is critical to offer soldiers quick and convenient healthy food options. Several strategies the Army could adopt to reduce the time to obtain healthy meals include providing soldiers with more access to healthy fresh options in establishments that currently exist on installations, such as grab-and-go options within DFAC, and improving the installation convenience store offerings of fresh fruits, healthy snacks and low-calorie beverages. The Army could also establish agreements with restaurants that provide healthy choices to be delivered to soldiers on their installation or at their worksite. It will also be beneficial to provide soldiers with instruction for healthy low-cost meal preparation in a military living or work environment.

### Strengths and limitations

During all phases of the qualitative evaluation study, our team set courses of action for recognized potential biases and limitations of the study. First, the present study was part of a larger evaluation of a WHPP, which covered all aspects of the RE-AIM framework, the multiple programme components and multiple behaviours. Although the focus group guides were comprehensive, they were not developed with an intention to focus specifically on military food environments. However, we explored themes related to the food environment as the topic naturally emerged within more than two-thirds of our focus group discussions. Second, the non-probability sampling technique used to gather participants for the study limits the generalizability of the findings to the overall population of active duty soldiers in the US Army. It is important to note that the number of focus groups (*n* 58) was sufficient to observe saturation of data within and between predetermined categories constructed from the RE-AIM framework. Moreover, the goal of qualitative inquiry in programme evaluation is not generalizability but rather a contextual understanding of how and why a programme was or was not effective^(^ ^44^ ^)^.

Third, the focus group methodology utilized may have introduced dominant respondent, overstatement and social acceptance biases. Based on the military hierarchical rank structure, power differentials can be problematic and pose concern if junior-ranking soldier participants are grouped with soldiers of more senior ranks^(^ ^28^ ^)^. We attempted to minimize these biases by segmenting focus groups by rank and role. Furthermore, the involvement of multiple independent analysts in drawing conclusions from the data maximized rigour and reduced subjectivity that could potentially be introduced by the evaluation team.

The evaluation team’s limited oversight of the battalion leaderships’ recruitment strategies may have introduced selection biases to the study. It is unknown whether battalion leaders strictly adhered to inclusion requirements, thus it is conceivable soldiers may have been selected for focus group participation based on their health status or level of WHPP engagement. However, as access to the soldiers is contingent upon military leadership involvement, battalion leadership buy-in and support for the focus groups were key to their effective recruitment and execution. If soldiers were selected for their high WHPP participation, their experience of barriers is likely similar to other soldiers on their installation because of similar work locations and shift schedules.

## Conclusions

Although a few focus group participants noted barriers to healthy eating while deployed or in field settings, the present study focused largely on the barriers in the campus-style setting where the WHPP was implemented (i.e. the garrison). Most existing research on nutrition and military personnel surrounds the rigidity of their schedules coupled with limited availability of food options in the field; however, barriers to healthy nutrition on the installation in the campus-style environments are often less examined. Scientific evidence on military nutrition programme planning on installations generally focuses nutrition strategies on physiological outcomes (e.g. weight loss) rather than the factors affecting the behaviour change (e.g. the significance of the food environment). Our evaluation of soldiers’ perceptions of their installations’ food environment and the extent to which it serves as a barrier to healthy eating resulted in piloting some of our recommendations in another WHPP. As suggested in nutrition intervention studies of a similar age group (e.g. 18–24 years)^(^ ^29^ ^,^ ^30^ ^)^ and campus-style setting, we found it imperative that nutrition messaging for our military nutrition WHPP appeal to young adult males by recognizing and incorporating gender-specific values and interests^(^ ^45^ ^)^. Furthermore, our results bolster advocacy for military worksite nutrition programming to be grounded in an ecological model with a strong emphasis on the environment and suggest more research on the behavioural effects from installation food options. No one strategy alone will change soldiers’ behaviours, but a combination of education, improved access to nutritious options and policy changes can work in concert to establish a food environment in which healthy choices become the natural and normative option. Continued work, intervention, research and evaluation in this area are imperative for the nation to maintain a healthy and ready fighting force.
